# Supplementary material for: Tropical peatlands and their conservation are important in the context of COVID-19 and potential future (zoonotic) disease pandemics
Source: PeerJ. 2020 Nov 17;8:e10283. doi: 10.7717/peerj.10283 (PMC7678489; doi:10.7717/peerj.10283)
Supplement: Supplemental Information 1 [file peerj-08-10283-s001.docx]

**Table S1. Structured literature review approach.**

| Databases searched | Google Scholar, Web of Science, Science Direct, Scopus, PubMed |
| --- | --- |
| Search string | ((“emerging infectious disease” OR “EID” OR “COVID-19” OR “SARS-CoV-2” OR “corona virus” OR “coronavirus”) AND (“tropical” AND (“peat” OR “peatland” OR “peatswamp” OR “peat-swamp”))  Science Direct only:  (("peat") AND ("emerging infectious disease" OR "EID" OR "COVID-19" OR "SARS-CoV-2" OR "corona virus" OR "coronavirus"))^1^ |
| Inclusion criteria | Article must explicitly look at the first half of the search operator string in relation to the second; i.e., be a EID study conducted in a tropical peatland area or be specifically looking at impacts of EIDs in a tropical peatland area |
| Exclusion criteria | Studies not in or concerning tropical peatlands, and studies discussing diseases that are ubiquitous (e.g., malaria) or non-zoonotic diseases (e.g. diseases caused by malnutrition), or that are otherwise not relevant in the context of EIDs according to the WHO (2014) definition: “An emerging infectious disease is one that either has appeared and affected a [human] population for the first time, or has existed previously but is rapidly spreading, either in terms of the number of people getting infected, or to new geographical areas”. |
| No. articles searched | First 300 articles, ordered by relevance. |

^1^ Adjusted and shortened for Science Direct owing to limits on the number of AND and OR operators permitted.

**References Cited**

WHO. 2014. *A Brief Guide to Emerging Infectious Diseases and Zoonoses.* World Health Organization. <https://apps.who.int/iris/handle/10665/204722>
