# Supplementary material for: Tropical peatlands and their conservation are important in the context of COVID-19 and potential future (zoonotic) disease pandemics
Source: PeerJ. 2020 Nov 17;8:e10283. doi: 10.7717/peerj.10283 (PMC7678489; doi:10.7717/peerj.10283)
Supplement: Supplemental Information 2 [file peerj-08-10283-s002.docx]

**Table S2. Structured literature search results.**

| **Database** | **No. hits** | **Reference** | **Key information** |
| --- | --- | --- | --- |
| Google Scholar | 2,270 | Hilser (2011) | Sampled gastro-intestinal parasites of 3 primate species in TPSF of Sebangau, Central Kalimantan, identifying “a rich diversity of parasite taxa”, including species found in humans. |
|  |  | Kanniah et al. (2020) | Suggested in previous studies that air pollution impacts COVID-19 vulnerability and mortality; mentions that tropical peatland fires are a contributor towards air pollution in SEA; finds air pollution lower during COVID-19 lockdowns. |
|  |  | Madinah et al. (2011) | Sampled 3 small mammal species from TPSF in Malaysia; found 5 species of ecto-parasites on these, one of which of medical importance (*Ixodes granulatus*). |
|  |  | Madinah et al. (2014) | Sampled small mammal ecto-parasites in Malaysia, including from TPSF areas; assesses parasite-host interaction networks; 21% of the 47 ecto-parasite species recorded are of medical importance. |
| Web of Science | 0 | - | - |
| PubMed | 0 | - | - |
| Science Direct | 288 | Kanniah et al. (2020) | Duplicate – see above. |
| Scopus | 562 | Bai et al. (2012) | Surveyed 19 bat species from Loreto (a TPSF region) in Peru; *Bartonella* bacteria samples cultured (genus has potential for zoonotic transmission and has public health significance); found infection rates 0-100% across bat species, with more than half of sampled animals infected. |
|  |  | Kanniah et al. (2020) | Duplicate – see above. |
|  |  | Nurcahyo, Konstanzová & Foitová (2017) | Total 51 parasite taxa known to infect wild, semi-wild and captive orangutans, including species reported found in humans; orangutan populations reviewed include those from TPSF areas; conclude orangutans in captivity can be infected with numerous parasites by humans and potential for zoonosis exists. |
| **Total no. included references = 6** | | | |

**References Cited**

Bai Y, Recuenco S, Gilbert AT, Osikowicz LM, Gómez J, Rupprecht C, Kosoy MY. 2012. Prevalence and diversity of *Bartonella* spp. in bats in Peru. *The American Journal of Tropical Medicine and Hygiene* 87:518-523. DOI: 10.4269/ajtmh.2012.12-0097

Hilser H. 2011. An Assessment of Primate Health in the Sabangau Peat-Swamp Forest, Central Kalimantan, Indonesian Borneo. MSc thesis, Oxford Brookes University, UK.

Kanniah KD, Kamarul Zaman NAF, Kaskaoutis DG, Latif MT. 2020. COVID-19's impact on the atmospheric environment in the Southeast Asia region. *Science of The Total Environment* 736:139658. DO: 10.1016/j.scitotenv.2020.139658

Madinah A, Abang F, Mariana A, Abdullah MT, Mohd-Azlan J. 2014. Interaction of ectoparasites-small mammals in tropical rainforest of Malaysia. *Community Ecology* 15:113. DOI: 10.1556/comec.15.2014.1.12

Madinah A, Fatimah A, Mariana A, Abdullah MT. 2011. Ectoparasites of small mammals in four localities of wildlife reserves in Peninsular Malaysia. *Southeast Asian Journal of Tropical Medicine and Public Health* 42:803-813

Nurcahyo W, Konstanzová V, Foitová I. 2017. Parasites of orangutans (primates: ponginae): An overview. *American Journal of Primatology* 79:e22650. DOI: 10.1002/ajp.22650
